# Metabolomics Analysis on Obesity-Related Obstructive Sleep Apnea After Weight Loss Management: A Preliminary Study

**DOI:** 10.3389/fendo.2021.761547

**Published:** 2022-01-03

**Authors:** Weijun Huang, Anyuan Zhong, Huajun Xu, Chong Xu, Anzhao Wang, Fan Wang, Xinyi Li, Yupu Liu, Jianyin Zou, Huaming Zhu, Xiaojiao Zheng, Hongliang Yi, Jian Guan, Shankai Yin

**Affiliations:** ^1^ Department of Otorhinolaryngology Head and Neck Surgery and Otolaryngology Institute of Shanghai Jiao Tong University and Shanghai Key Laboratory of Sleep Disordered Breathing, Shanghai Jiao Tong University Affiliated Sixth People’s Hospital, Shanghai, China; ^2^ Department of Respiratory Diseases, The Second Affiliated Hospital of Soochow University, Suzhou, China; ^3^ Center for Translational Medicine and Shanghai Key Laboratory of Diabetes Mellitus and Shanghai Key Laboratory of Sleep Disordered Breathing, Shanghai Jiao Tong University Affiliated Sixth People’s Hospital, Shanghai, China

**Keywords:** obstructive sleep apnea, metabolomics, Roux-en-Y gastric bypass, obesity, type 2 diabetes

## Abstract

**Objective:**

Roux-en-Y gastric bypass (RYGB) surgery is an effective type of weight loss management and may improve obesity-related obstructive sleep apnea (OSA). Obese subjects who meet the criteria for surgery with OSA were enrolled. We investigated the metabolomic effects of RYGB on OSA.

**Methods:**

Clinical data, serum measurements including indices of glycolipid metabolism, and polysomnography (PSG) measurements were collected at baseline and 6 months after RYGB surgery. Metabolomic analysis was performed using ultra-performance liquid chromatography-mass spectrometry.

**Results:**

A group of 37 patients with obesity, type 2 diabetes (T2DM) and suspected OSA were enrolled of which 27 were OSA subjects. After RYGB surgery, metabolic outcomes and sleep parameters were all significantly improved. The OSA remission group had lower valine, isoleucine, and C24:1(cis-15) levels, and higher trimethylamine N-oxide, hippurate, and indole-3-propionic acid levels after RYGB surgery. A combination of preoperative indices (age, apnea-hypopnea index (AHI), fasting C-peptide level, and hippurate level) predicted the RYGB effect size in obese patients with T2DM and OSA, with an area under receiver operating characteristic curve of 0.947, specificity of 82.4%, and sensitivity of 100%.

**Conclusions:**

RYGB surgery may significantly improve the metabolic status of patients with obesity, T2DM and OSA. A combination of preoperative indices (age, AHI, fasting C peptide level, and hippurate level) may be useful for predicting the effect size of RYGB in obese patients with T2DM and OSA. The mechanisms underlying OSA remission need to be explored.

## Introduction

Obstructive sleep apnea (OSA) has a high prevalence and is characterized by recurrent episodes of upper airway obstruction during sleep. The resulting abnormal breathing leads to intermittent hypoxia and sleep fragmentation. OSA increases all-cause mortality (1, 2) by further progression of pathological changes involving sympathetic activation, oxidative stress, inflammation, exaggerated negative intrathoracic pressure, insulin resistance, endothelial dysfunction, or other factors related to OSA such as excessive daytime sleepiness, obesity and lung disease (2, 3). All of these in turn contribute to the pathogenesis of cardiovascular diseases, metabolic dysregulation, and other systemic disorders and consequently threaten patients’ lives (4). In total, 120 million people are at high risk of OSA in China (5), which has a prevalence exceeding 5% (5). These subjects are withstanding the adverse effects causing by OSA. Thus, OSA is not only a serious health problem but also a socioeconomic issue (6, 7).

﻿OSA patients are more likely to be obese and have type 2 diabetes (T2DM). As a bariatric surgery technique, Roux-en-Y gastric bypass (RYGB) surgery is an effective type of weight loss management which can improve or resolve obesity-related comorbidities such as T2DM (8, 9), and may also improve accompanied OSA. RYGB surgery can resolve abnormal sleep architecture and restore normal hypoxic oxygen status (10–13). Former study revealed serum metabolites changes of OSA patients after multilevel sleep surgery (14). However, the serum metabolites changes of OSA patients who have undergone RYGB remains unclear.

Recently, metabolomics, a novel tool for exploring the physiological and pathological mechanisms underlying disease, has been used to identify new biomarkers by exploring the correlation between the biochemical reactions of small molecules in cells and the internal state of the body (14–17). Previous studies revealed that free fatty acids, acylcarnitines, amino acids, bile acids, and lipid species could predict T2DM remission after bariatric surgery more effectively than existing prediction models (18, 19). Thus, understanding the characteristic serum metabolites associated with OSA after RYGB is important for finding potential pathways underlying and may be helpful in developing strategies for intervention or efficacy prediction.

In this study, we performed targeted metabolomics, which provided quantitative data on 52 metabolites (mainly fatty acids and amino acids), to assess metabolomic changes after RYGB, and to identify biomarkers and pathways that are helpful for predicting the progress and prognosis of OSA.

## Materials and Methods

### Patients

This study was conducted in accordance with the Declaration of Helsinki and was approved by the Ethics Committee. All the participants signed informed consent forms. The surgeons followed the latest guidelines for metabolic surgery (20–23). The inclusion criteria were a T2DM duration ≤ 15 years with adequate islet function (defined by C peptide release test when a fasting C-peptide level > 1 ng/mL and a peak value > 2 ng/mL); age of 16–65 years; and body mass index (BMI) > 35 kg/m^2^, or BMI of 25–27.5 kg/m^2^ with poorly controlled T2DM and more than two symptoms of metabolic syndrome, or T2DM complications and a BMI > 27.5 kg/m^2^ with poorly controlled T2DM. The exclusion criteria were systemic disease, such as pulmonary, renal, liver, cardiovascular, or neurological disease incompatible with surgery; incomplete polysomnography (PSG) data; previous OSA treatment (e.g., surgery, continuous positive airway pressure, oral appliance therapy); current therapy that might affect the clinical and metabolomic results (e.g., hormone replacement therapy); previous open abdominal surgery; acute T2DM complications, type 1 diabetes, or secondary diabetes as a consequence of endocrine disease, hereditary disease or medication (e.g., pancreatectomy, Cushing’s syndrome); and a mental disorder or severe alcohol or drug dependency. Mortality and severe complications did not occur in any patients. They were all given nutritional guidance after RYGB surgery and ﻿followed-up for 6 months.

### Clinical and Biochemical Measurements

Anthropometric parameters including height, weight, circumference of the waist and hip were recorded. Systolic blood pressure (SBP) and diastolic blood pressure (DBP) were measured by a standard sphygmomanometer. The mean values of two consecutive measurements were recorded before PSG, as previously described (11). BMI was calculated as weight/height^2^. At 7 AM, fasting blood and urine samples were collected from all patients who underwent PSG. Indices of glycolipid metabolism and the lipid profile, including fasting blood glucose, fasting insulin, insulin (120 min), fasting C-peptide, C-peptide (120 min), glycated hemoglobin (GHb), glycated albumin (GA), cholesterol (TC), triglyceride (TG), high-density lipoprotein (HDL), low-density lipoprotein (LDL), apolipoprotein (Apo) A-1, ApoB, ApoE, and lipoprotein (a) (Lp(a)), were measured in the clinical laboratory of our hospital. The homeostasis model assessment of insulin resistance (HOMA-IR) was calculated as fasting insulin × fasting plasma glucose/22.5 (24).

### Sleep Evaluation

Patients were suspected OSA subjects and underwent portable PSG to assess nocturnal sleep and determine OSA status. Nasal airflow, thoracic/abdominal movement, pulse oximetry, body posture, and snoring were recorded continuously overnight (10 PM–6 AM). Two skilled technicians checked and analyzed the data. The PSG parameters consisted of the apnea-hypopnea index (AHI), oxygen desaturation index (ODI), pulse oxygen saturation (SpO2), lowest pulse oxygen saturation (LSpO2), and microarousal index (MAI). PSG was performed at baseline and 6 months after RYGB surgery. Before the PSG sleep study, daytime sleepiness was assessed using the Epworth Sleepiness Scale (ESS). OSA was classified according to the American Academic Sleep Medicine 2007 criteria (25). Patients with an AHI ≥ 5 events/h were diagnosed with OSA. OSA remission was defined as an AHI > 5 events/h before surgery and an AHI < 5 events/h after surgery. In contrast, patients with AHI > 5 events/h both pre- and postoperatively were classified were classified as OSA non-remission.

### ﻿Surgical Procedure

The RYGB was performed laparoscopically by the same team using a standardized method (26). A 25-mL gastric pouch was divided from the distal remnant. The biliopancreatic and alimentary limbs were 100–120 cm in length. After completion of the surgical procedures, the patients were further evaluated and monitored, and were discharged to follow-up once they were stable.

### Statistical Analyses

Normal distributed continuous variables are shown as the mean ± standard deviation and non-normal distributed continuous variables are shown as the median (first to third quartile). Categorical variables are shown as percentages. The Shapiro-Wilk test was performed to assess the normality of the data. According to the results of the normality test, the paired Student’s *t*-test or Wilcoxon rank-sum test was performed for comparison of parameters before and after surgery. Analysis of variance or the Kruskal–Wallis test was used to compare multiple groups. We performed logistic regression analysis to identify significantly altered metabolites and confounding variables, controlling for potential confounders such as sex, BMI, fasting glucose, fasting insulin, medication for T2DM, hypertension and dyslipidaemia, ESS, and LSpO2. Using the PSG results as the gold standard, the accuracy of the prediction model was determined by receiver operating characteristic (ROC) curve analysis, i.e., the area under the curve (AUC), with the sensitivity and specificity calculated according to the best diagnostic cut-off points. p < 0.05 was considered statistically significant. All analyses were performed using SPSS (version 23.0; SPSS Inc., Chicago, IL, USA, 2015) and R software (version 3.6.1; R Development Core Team, Vienna, Austria). All subjects were required to go to bed at a certain time and to stop smoking and drinking alcohol during the follow-up study period. Two skilled physicians independently entered and analyzed the data to ensure the accuracy thereof. Patients who were lost to follow-up or had incomplete data were excluded from the analysis.

### Metabolomic Analyses

Metabolomics provides detailed information on molecular structure and can detect a wide range of metabolites simultaneously (27). ﻿The metabolomics kit used in this investigation was also used in our previous study (28), and provided quantitative data on 52 metabolites, which were mainly fatty acids and amino acids. An unsupervised dimensionality reduction method, namely principal component analysis, was used to investigate the internal characteristics of the dataset and eliminate singularities. Supervised partial least squares-discriminant analysis (PLS-DA) and orthogonal partial least squares-discriminant analysis (OPLS-DA) were performed to visualize the metabolic differences between the groups. The variable importance in the projection (VIP) score reflects the importance of each variable in the model according to their overall contribution. Significantly altered metabolites (p < 0.05) with a VIP score ≥ 2 in OPLS-DA models were selected. Metabolites confirmed by ultra-performance liquid chromatography-mass spectrometry, and those that were statistically significant with a non-zero correlation with OSA biomarkers, were analyzed by logistic regression.

## Results

### Anthropometric and Clinical Information

**Figure 1:**
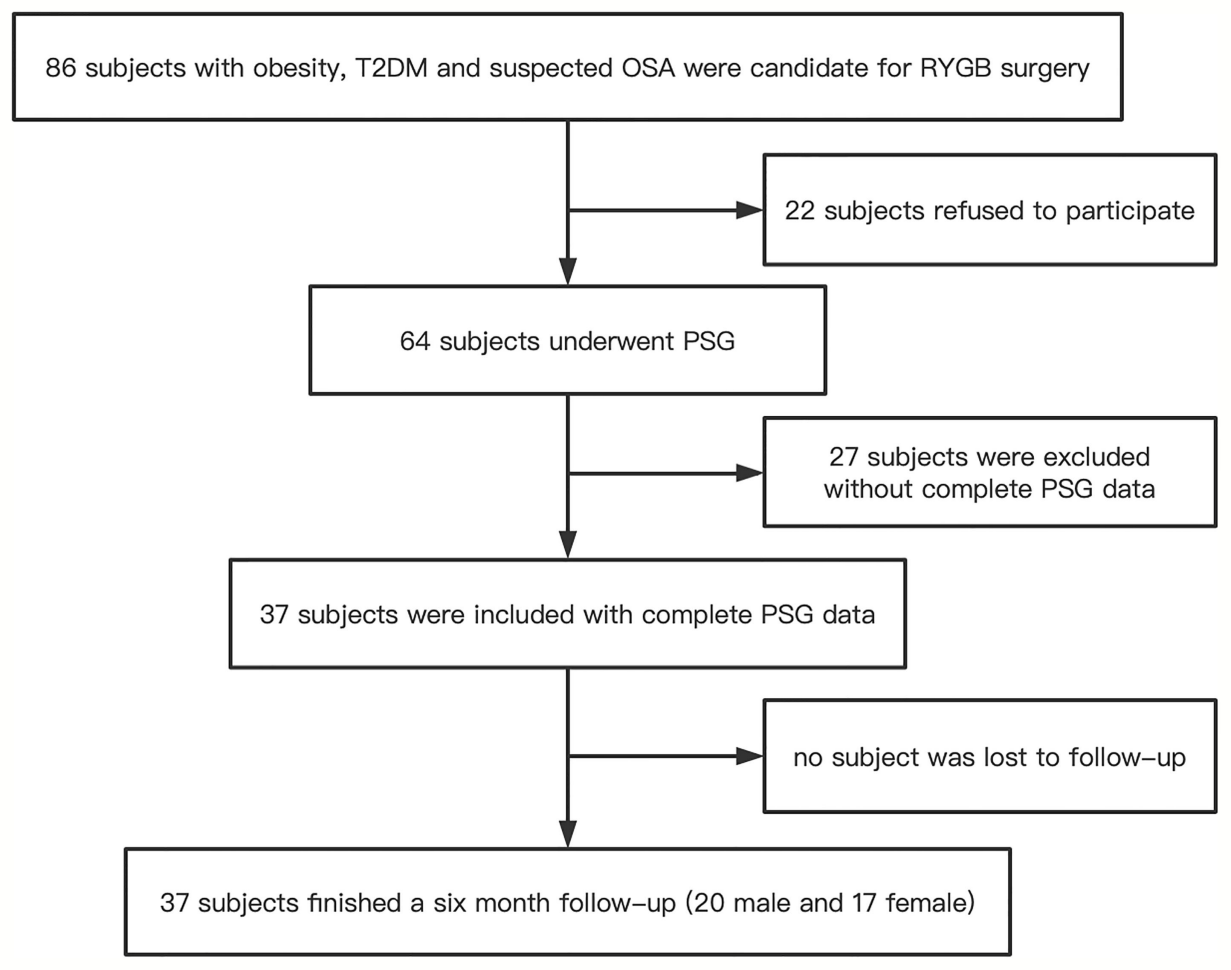
Flow diagram of the recruitment process. T2DM, type 2 diabetes; OSA, obstructive sleep apnea; RYGB, Roux-en-Y gastric bypass surgery; PSG, polysomnography.

Of the 86 subjects with obesity, T2DM and suspected OSA who were candidates for RYGB surgery, 22 refused to participate. After 64 subjects who underwent PSG, 27 were excluded due to incomplete PSG data. Finally, 37 subjects (20 men and 17 women) with complete PSG data were included, and they all completed the 6-month follow-up (**Figure 1**). The anthropometric and clinical characteristics at baseline and 6 months after RYGB surgery are shown in **Table 1**. Almost all anthropometric, clinical, and sleep variables improved after RYGB surgery. As shown in **Table 1**, waist circumference, hip circumference, waist-to-hip ratio, BMI, SBP, DBP, fasting glucose, fasting insulin, insulin (120 min), fasting C-peptide, C-peptide (120 min), HOMA-IR, GHb, GA, TC, TG, LDL, ApoB, ApoE, ESS, AHI, and ODI were decreased, whereas HDL, mean SpO2, and LSpO2 were increased. ApoA-1, Lp(a), and MAI showed no significant changes. According to postoperative AHI, OSA patients were then divided into two subgroups: OSA remission and OSA non-remission. The aforementioned parameters were similar between the subgroups showing significant improvement in obesity and OSA severity, except that the postoperative AHI was > 5 events/h in the non-remission group (**Table 2**). In further subgroup analyses with decrease rate of AHI after RYGB surgery, the aforementioned parameters were improved significantly as well. Compared to the group with decrease rate of AHI ≤ 70% after RYGB surgery, the AHI, MAI, fasting glucose, and GA were lower in the group showing a decrease rate of AHI > 70% after RYGB surgery, whereas decrease value of AHI and decrease rate of AHI were higher (**Supplementary Table S1**). Better improvement in glucose metabolism was accompanied with better OSA improvement.

**Table 1:** Anthropometric and clinical characteristics of the enrolled patients.

Characteristics	Before RYGB surgery (n = 37)	After RYGB surgery (n = 37)
**Males, n (％)**	20 (54.05)	
**Age, y**	43.97 ± 12.70	
**Follow-up time, m**	6.03 ± 0.10	
**Medication for T2DM, n (％)**	35 (94.59)	
**Medication for hypertension, n (％)**	22 (59.46)	
**Medication for dyslipidaemia, n (％)**	16 (43.24)	
**Waist circumference, cm**	105.56 ± 12.17	86.73 ± 8.41***
**Hip circumference, cm**	108.19 ± 8.85	96.64 ± 6.49***
**Waist-to-hip ratio**	0.97 ± 0.06	0.90 ± 0.05***
**BMI, kg/m^2^**	31.96 ± 3.63	24.62 ± 2.91***
**Reductions of BMI, kg/m^2^**	7.34 ± 2.31	
**SBP, mmHg**	130 (122-150)	120 (110-122)***
**DBP, mmHg**	85 (80-93)	77 (70-80)***
**Fasting glucose, mmol/L**	7.66 (6.42-9.07)	5.43 (4.69-6.06)***
**Fasting insulin, µU/mL**	16.92 (11.36-30.80)	8.22 (4.70-9.98)***
**Insulin (120 min), µU/mL**	75.80 (49.31-157.55)	17.42 (9.89-30.37)***
**Fasting C peptide, ng/mL**	3.05 (2.32-3.39)	2.03 (1.73-2.29)***
**C peptide (120 min), ng/mL**	6.13 (3.59-11.60)	4.91 (3.52-7.51)*
**HOMA-IR**	5.60 (3.61-11.06)	1.86 (1.12-2.92)***
**GHb, ％**	7.60 (6.20-8.60)	5.80 (5.35-6.20)***
**GA, ％**	17.72 (16.00-19.65)	14.00 (12.65-15.15)***
**TC, mmol/L**	4.99 (4.49-5.64)	4.18 (3.68-4.76)***
**TG, mmol/L**	1.70 (1.27-2.38)	0.97 (0.74-1.22)***
**HDL, mmol/L**	1.02 (0.90-1.14)	1.21 (1.09-1.32)***
**LDL, mmol/L**	3.11 (2.49-3.51)	2.33 (2.05-2.75)***
**ApoA-1, g/L**	1.05 (0.97-1.11)	1.07 (1.01-1.13)
**ApoB, g/L**	0.92 (0.86-1.05)	0.68 (0.61-0.73)***
**ApoE, mg/dL**	5.02 (4.32-5.51)	3.58 (3.12-4.02)***
**Lp(a), mg/dL**	16.54 (12.40-19.90)	16.60 (10.93-19.72)
**ESS**	8 (6-10)	3 (2-6)***
**ESS>10, n (%)**	9 (24.3)	0 (0)***
**AHI, events/h**	12.70 (4.35-28.60)	3 (2.15-7)***
**LSpO2, %**	81 (76-87)	89 (87-91)***
**Mean SpO2, %**	94 (92-95)	96 (94-97)***
**ODI, events/h**	16.60 (5.10-29.70)	5 (1.45-7)***
**MAI, events/h**	16.80 (12.55-19.05)	17.10 (10.15-21.65)

BMI, body mass index; SBP, systolic blood pressure; DBP, diastolic blood pressure; HOMA-IR, homeostasis model assessment of insulin resistance; GHb, glycated hemoglobin; GA, glycated albumin; TC, total cholesterol; TG, triglyceride; HDL, high-density lipoprotein cholesterol; LDL, low-density lipoprotein cholesterol; ApoA-I, apolipoprotein A-I; ApoB, apolipoprotein B; ApoE, apolipoprotein E; Lp(a), lipoprotein (a); ESS, Epworth sleepiness score; AHI, apnea–hypopnea index; LSpO2, lowest pulse oxygen saturation; SpO2, pulse oxygen saturation; ODI, oxygen desaturation index; MAI, micro-arousal index. P-value indicates significant differences before and after RYGB surgery. * indicated p-value ＜ 0.05 before versus after surgery in subgroup analysis. *** indicated p-value ＜ 0.001 before versus after surgery in subgroup analysis.

**Table 2:** Clinical characteristics of the OSA remission and non-remission groups before and after RYGB surgery.

	OSA remission (n = 17)			OSA non-remission (n = 10)	
Characteristics	Before RYGB surgery		After RYGB surgery	Before RYGB surgery	After RYGB surgery
Males, n (%)	10 (58.82)			4 (40)	
Age, y	40.06 ± 11.09			54.90 ± 10.60^a^	
Follow-up time, m	6.04 ± 0.14			6.02 ± 0.08	
Medication for T2DM, n (%)	16 (94.12)			10 (100)	
Medication for hypertension, n (%)	13 (76.47)			5 (50)	
Medication for dyslipidaemia, n (%)	7 (41.18)			4 (40)	
Waist circumference, cm	107.39 ± 14.18	86.04 ± 8.84***		108.20 ± 12.23	88.97 ± 9.33***
Hip circumference, cm	109.22 ± 9.35	96.45 ± 6.50***		110.90 ± 9.70	98.06 ± 8.22**
Waist-to-hip ratio	0.98 ± 0.07	0.89 ± 0.05***		0.97 ± 0.05	0.91 ± 0.03**
BMI, kg/m^2^	32.64 ± 3.88	24.76 ± 3.18***		32.61 ± 3.79	25.10 ± 3.27***
Reductions of BMI, kg/m^2^	7.89 ± 2.25			7.51 ± 1.72	
SBP, mmHg	136 (120-154)	120 (110-130)**		147 (130-154)	120 (110-126)***
DBP, mmHg	82 (80-90)	80 (72-85)		93 (84-100)	70 (70-81)***
Fasting glucose, mmol/L	7.49 (6.03-8.95)	5.11 (4.58-5.64)***		7.60 (6.43-8.87)	5.84 (5.09-6.41)**^b^
Fasting insulin, µU/mL	16.92 (12.60-30.92)	6.68 (4.20-9.28)***		21.55 (10.29-41.98)	9.02 (6.52-11.99)**
Insulin (120 min), µU/mL	77.32 (48.98-161.50	14.64 (9.44-34.86)***		99.84 (52.11-138.58)	23.17 (12.27-39.74)**
Fasting C peptide, ng/mL	3.17 (3.03-4.37)	2.01 (1.59-2.46)***		2.37 (1.40-3.52)^a^	2.03 (1.71-2.33)
C peptide (120 min), ng/mL	9.10 (5.75-11.94)	4.91(3.53-6.70)**		4.22 (2.27-11.38)	4.23 (3.67-7.98)
HOMA-IR	5.60 (3.77-11.88)	1.28 (0.91-2.38)***		7.91 (2.99-14.67)	2.09 (1.51-3.51)**
GHb, %	7.10 (6.00-8.95)	5.40 (5.05-5.96)***		7.55 (6.83-9.45)	6.20 (5.79-6.70)**^b^
GA, %	17.20 (13.70-19.75)	13.20 (11.60-14.80)***		18.21 (16.75-20.98)	15.20 (12.38-18.03)**^b^
TC, mmol/L	4.67 (3.97-5.06)	4.37 (3.79-4.92)		5.00 (4.32-5.89)	3.98 (3.60-4.28)***
TG, mmol/L	1.45 (1.25-3.14)	0.82 (0.72-1.19)**		1.66 (1.26-2.53)	1.05 (0.88-1.31)**
HDL, mmol/L	1.00 (0.90-1.03)	1.21 (1.11-1.27)***		1.14 (0.87-1.16)	1.13 (1.00-1.46)
LDL, mmol/L	2.96 (2.31-3.14)	2.62 (2.09-3.16)		3.27 (1.98-3.63)	2.19 (2.01-2.27)***
ApoA-1, g/L	1.06 (0.99-1.13)	1.07 (1.01-1.09)		1.13 (1.05-1.29)	1.07 (0.97-1.12)
ApoB, g/L	0.90 (0.87-0.93)	0.69 (0.56-0.73)***		0.93 (0.77-1.07)	0.69 (0.59-0.76)**
ApoE, mg/dL	5.10 (4.21-5.91)	3.66 (2.71-4.12)***		5.20 (4.72-5.97)	3.78 (2.99-4.87)**
Lp(a), mg/dL	16.47 (11.30-21.31)	16.60 (10.12-20.17)		16.47 (11.40-23.58)	16.60 (12.08-22.56)
ESS	9 (6-15)	3 (2-6)***		10 (7-13)	4 (3-6)**
ESS>10, n (%)	3 (17.65)	0***		3 (30.00)	0***
AHI, events/h	19.25 (13.20-21.20)	2.76 (1.60-2.88)***		29.65 (19.25-44.15)^a^	8.85 (6.94-18.43)***^b^
Decrease value of AHI, events/h	16.48 (8.25-37.36)			16.04 (7.13-28.58)^b^	
Decrease rate of AHI, %	85.64 (82.84-88.55)			64.86 (26.67-70.40)^b^	
LSpO2, %	81 (78-85)	88 (87-91)***		78 (69-81)	87 (81-90)**^b^
Mean SpO2, %	94 (92-95)	96 (95-97)***		93 (92-94)	96 (94-97)**
ODI, events/h	20.55 (12.80-21.90)	3.00 (1.30-6.02)***		33.15 (21.55-44.75)	7.05 (5.67-17.63)^b^
MAI, events/h	17.83 (13.15-21.42)	17.82 (11.45-19.25)		18.32 (14.48-26.38)	17.82 (10.08-27.40)

BMI, body mass index; SBP, systolic blood pressure; DBP, diastolic blood pressure; HOMA-IR, homeostasis model assessment of insulin resistance; GHb, glycated hemoglobin; GA, glycated albumin; TC, total cholesterol; TG, triglyceride; HDL, high-density lipoprotein cholesterol; LDL, low-density lipoprotein cholesterol; ApoA-I, apolipoprotein A-I; ApoB, apolipoprotein B; ApoE, apolipoprotein E; Lp(a), lipoprotein (a); ESS, Epworth sleepiness score; AHI, apnea–hypopnea index; LSpO2, lowest pulse oxygen saturation; SpO2, pulse oxygen saturation; ODI, oxygen desaturation index; MAI, microarousal index. ** indicated p-value ＜ 0.05 before versus after surgery in subgroup analysis. *** indicated p-value ＜ 0.001 before versus after surgery in subgroup analysis. ^a^indicated the p-value for the difference between the OSA remission and non-remission groups before surgery. ^b^indicated the p-value for the difference between the OSA remission and non-remission groups after surgery.

### Differences in Baseline Metabolomic Profiles Between the OSA Remission and Non-Remission Groups

**Figure 2:**
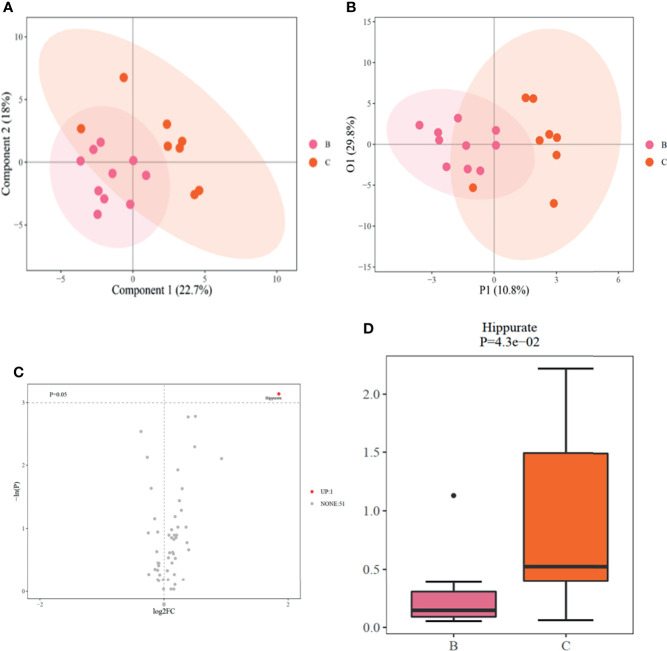
**(A).** PLSDA scores between OSA non-remission and remission groups at baseline; **(B)**. OPLS-DA scores between OSA non-remission and remission groups at baseline; **(C)**. Volcano plot of differential metabolites generated by univariate analysis between OSA non-remission and remission groups at baseline. UP indicates that the level of the metabolite (hippurate) was higher in the OSA non-remission group than the OSA remission group before RYGB surgery. NONE indicates 51 metabolites with no significant difference between the OSA remission and non-remission groups before RYGB surgery; **(D)**. Boxplot of hippurate generated by univariate analysis compared between OSA non-remission and remission groups. **(B)** indicates the OSA remission group and **(C)** indicates the OSA non-remission group. PLS-DA, partial least squares-discriminant analysis; OPLS-DA, orthogonal partial least squares-discriminant analysis; OSA, obstructive sleep apnea; RYGB, Roux-en-Y gastric bypass surgery.

The normalized data of the 52 quantified metabolites are shown in **Supplementary Table S2**. The levels of 38 metabolites were lower, and those of 14 were higher, in the OSA remission group compared to the non-remission group at baseline. Differences in metabolomic profiles between the OSA non-remission and remission groups at baseline were demonstrated by PLS-DA and OPLS-DA (**Figures 2A, B**). Univariate analyses demonstrated that hippurate was a potential biomarker, with a VIP score ≥ 2 and p < 0.05 (**Figures 2C, D**).

### Identification of Significantly Altered Metabolites Associated With OSA Remission

**Figure 3:**
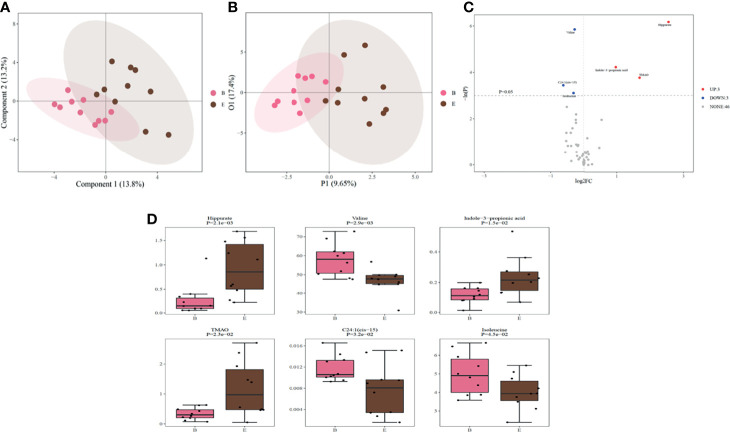
**(A).** PLS-DA scores reflecting metabolic changes in the OSA remission group before and after RYGB surgery; **(B)**. OPLS-DA scores reflecting metabolic changes in the OSA remission group before and after RYGB surgery. **(C)** Volcano plot of differential metabolites generated by the univariate analysis before and after RYGB surgery; UP indicates the three metabolites that increased after RYGB surgery in the OSA remission group. DOWN indicates the three metabolites that decreased after RYGB surgery in the OSA remission group. NONE indicates the 46 metabolites with no significant expression differences after RYGB surgery in the OSA remission group; **(D)** Boxplot of six differential metabolites with statistical significance generated by univariate analysis. B indicates the preoperative OSA remission group and E indicates the postoperative OSA remission group. PLS-DA, partial least squares-discriminant analysis; OSA, obstructive sleep apnea; OPLS-DA, orthogonal partial least squares-discriminant analysis; RYGB, Roux-en-Y gastric bypass surgery.

We analyzed the metabolite levels of patients with OSA remission before and after surgery (**Figures 3A, B**, and **Supplementary Table S3**). The valine, C24:1(cis-15) and isoleucine levels decreased after surgery (valine: from 58.174 [50.808–62.089] ng/mL to 47.821 [45.342, 49.661] ng/mL [p = 0.003]; C24:1(cis-15): from 0.011 [0.010, 0.013] ng/mL to 0.008 [0.003, 0.010] ng/mL [p = 0.032]; isoleucine: from 4.908 [3.999, 5.794] ng/mL to 3.937 [3.561, 4.613] ng/mL [p = 0.045]), whereas the hippurate, trimethylamine N-oxide (TMAO), and indole-3-propionic acid levels increased (hippurate: from 0.146 [0.091, 0.306] ng/mL to 0.856 [0.503, 1.420] ng/mL [p = 0.002]; TMAO: from 0.303 [0.203, 0.473] ng/mL to 0.972 [0.475, 1.815] ng/mL [p = 0.023]; indole-3-propionic acid: from 0.110 [0.083, 0.159] ng/mL to 0.216 [0.148, 0.270] ng/mL [p = 0.015]) (**Figures 3C, D**, and **Table 3**). Heatmaps based on the Spearman correlation matrix demonstrated clear correlations between the clinical indicators and metabolomic profiles (**Supplementary Figures S1**–**S3**). ﻿In the heatmaps, stronger correlations are displayed in darker colors. **Supplementary Figure S1** shows the correlations between clinical indicators and metabolomic profiles in all patients before and after RYGB, whereas **Supplementary Figure S2** shows the correlations in OSA patients. As shown in **Supplementary Figure S3**, changes in clinical parameters were significantly correlated with metabolomic profiles. The correlations among the mean SaO2, LSpO2, MAI, waist circumference, hip circumference, waist-to-hip ratio, BMI, SBP, DBP, fasting glucose, fasting insulin, insulin (120 min), C-peptide (120 min), HOMA-IR, GA, HDL, LDL, TC, ApoE, ESS, and certain metabolites were statistically significant in the OSA remission group.

**Table 3:** Metabolomic changes in the OSA remission group after surgery.

Class	Metabolite	Before RYGB surgery (ng/mL)	After RYGB surgery (ng/mL)	P
Amino acids	Valine	58.175 (50.808, 62.088)	47.821 (45.342, 49.661)	0.003
Amino acids	Isoleucine	4.908 (3.999, 5.794)	3.937 (3.561, 4.613)	0.045
MUFAs	C24:1(cis-15)	0.011 (0.010, 0.013)	0.008 (0.003, 0.010)	0.032
Organic nitrogen compounds	TMAO	0.303 (0.203, 0.473)	0.972 (0.475, 1.815)	0.023
Benzene and substituted derivatives	Hippurate	0.146 (0.091, 0.306)	0.856 (0.503, 1.420)	0.002
Indoles	Indole-3-propionic acid	0.110 (0.083, 0.159)	0.216 (0.148, 0.270)	0.015

MUFA, monounsaturated fatty acid; TMAO, trimethylamine N-oxide. p-value indicated significant differences in metabolomics before versus after RYGB surgery.

### Potential Metabolic Biomarkers of RYGB Effect Size for OSA

**Figure 4:**
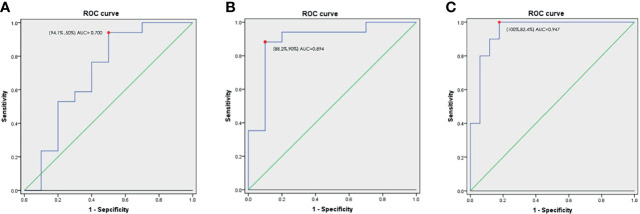
**(A).** ROC curve and AUC values of hippurate on predicting OSA remission after RYGB surgery; **(B)** ROC curve and AUC values of combined preoperative indices (age, AHI, and fasting C-peptide level) on predicting OSA remission after RYGB surgery; **(C)** ROC curve and AUC values of combined preoperative indices (age, AHI, and fasting C-peptide and hippurate levels) on predicting OSA remission after RYGB surgery. Abbreviations: ROC, receiver operating characteristic; AUC, area under the curve; AHI, apnea–hypopnea index.

We performed logistic regression and ROC analyses to determine the metabolites with potential as biomarkers. Compared to the non-remission patients, only hippurate level at baseline was significantly lower in the remission patients (VIP score > 2, p < 0.05; **Supplementary Table S2**). For hippurate, the sensitivity and specificity of the ROC curve to distinguish patients with obesity, T2DM and OSA who could versus could not achieve OSA remission were 94.1% and 50%, respectively. The AUC value was 0.700 (95% CI: 0.472–0.928; **Figure 4A**). We further used forward logistic regression to identify baseline clinical parameters predicting likelihood of OSA remission after RYGB surgery, age, AHI, and fasting C-peptide level were statically significant. When we adjusted the confounding factors of BMI, sex, fasting glucose and fasting insulin, the significance still existed (**Supplementary Table S4**). In a model comprising three preoperative clinical parameters (age, AHI, and fasting C-peptide level), the sensitivity and specificity were 88.2% and 90%, respectively. The AUC value was 0.894 (95% CI: 0.755–1.000; **Figure 4B**). We also established a model that included four preoperative variables (age, AHI, fasting C-peptide level, and hippurate level). The equation used to predict OSA remission is as follows:


$$\begin{array}{l} \ln\frac{p}{1-p}=\exp(9.876-0.194[age]-0.064[\text{preoperative AHI}]\\ +1.005[\text{preoperative fasting C peptide}]-3.757[\text{preoperative hippurate}]) \end{array}$$

p is the probability of achieving OSA remission after RYGB surgery. The specificity and sensitivity for distinguishing obese T2DM and OSA patients who could versus could not achieve remission were 82.4% and 100%, respectively. The AUC value was 0.947 (95% CI: 0.866–1) (**Figure 4C**). Patients who were younger, with a lower preoperative AHI and baseline level of hippurate, and a higher preoperative fasting C-peptide level, were more likely to achieve OSA remission after RYGB surgery.

## Discussion

To date, no study has assessed the metabolic profiles of patients with OSA after RYGB surgery in detail. Our study is the first to show that the levels of valine, isoleucine, and C24:1(cis-15) are decreased, whereas those of hippurate, TMAO, and indole-3-propionic acid are increased, after RYGB surgery compared to baseline in OSA patients in remission providing insight for future study on potential mechanisms. A combination of preoperative indices (age, AHI, and fasting C-peptide and hippurate levels) may be highly sensitive and specific for predicting the RYGB effect size for patients with obesity, T2DM and OSA. Since changes in the clinical parameters were significantly correlated with metabolomic profiles, our data also provide insight into the important mechanistic pathways of OSA (29).

Isoleucine and valine, which are branched chain amino acids (BCAAs), were decreased after RYGB surgery in our OSA remission group. Studies have shown that obese patients with T2DM have higher levels of isoleucine and valine than healthy individuals (30, 31). In addition, mitochondrial dysfunction may be caused by downstream accumulation of BCAA metabolites (32). Meanwhile, elevated BCAA levels are associated with insulin resistance (33), diabetes (34) and coronary artery disease (35), but the physiological mechanisms underlying the regulation of circulating BCAA concentrations remain unknown (36–39). Whether BCAAs are a causative factor in insulin resistance and T2DM or just a biomarker of impaired insulin action requires further study. Two potential mechanisms explaining how BCAAs might contribute to insulin resistance in obesity and T2DM have emerged (32). First, mammalian target of rapamycin complex 1 (mTORC1) signaling is activated by excess of dietary BCAAs leading to insulin resistance and T2DM. Second, increased levels of BCAAs are a biomarker of impaired metabolism and BCAA dysmetabolism also leads to the accumulation of toxic metabolites that cause mitochondrial dysfunction in pancreatic islet β cells (or elsewhere). These findings indicate that mitochondrial dysfunction may play a role in OSA pathophysiology. When OSA improves, mitochondrial function recovers, causing a decrease in BCAA levels. Further studies are needed to determine the potential regulatory roles of the metabolites identified herein in OSA (40).

C24:1(cis-15), i.e., nervonic acid, is an ultra-long chain monounsaturated fatty acid that was first discovered in the nerve tissue of mammals (41). Increased nervonic acid in the diet improves the metabolic parameters of mice fed a high-fat diet (42). Nervonic acid is negatively associated with HOMA-IR and coronary risk factors, and protects against poor obesity-related metabolic outcomes (43). Martin et al. (44) found that saturated fatty acid and nervonic acid levels in obese adolescents were higher than those in the controls. Also, greater weight loss is associated with larger decreases in nervonic acid levels (45). Therefore, after RYGB surgery, obesity improves, and nervonic acid requirements are reduced. In other words, high levels of nervonic acid are not needed to improve obesity and metabolism, and the postoperative nervonic acid level thus decreases. However, the functional roles of nervonic acid in obesity and OSA are not fully understood. Additional research is needed to determine whether changes in nervonic acid expression are involved in the improvement in the pathophysiology, or are simply a consequence of the metabolic and OSA improvements provided by RYGB.

Hippurate is formed by benzoic acid and glycine in the liver. The ability of liver mitochondria to synthesize hippurate is related to the supply of adenosine triphosphate, glycine, and coenzyme A. A significant increase in serum glycine and alanine at 6 months after RYGB surgery was found in this study; thus, hippurate levels increased while BCAA levels decreased (46, 47), leading to improvement in OSA, restoration of mitochondrial function, and increased synthesis of hippurate after RYGB surgery. In our OSA remission group, the remission may have been associated with mitochondrial unction, whereas in the non-remission group, some other factor may have accounted for the high levels of hippurate before surgery, and thus the non-resolution of OSA after surgery.

Lower preoperative TMAO reflects milder inflammation status, making OSA remission more likely. Animal and clinical studies (15) have demonstrated that OSA is correlated with changes in intestinal microbes. Tremaroli et al. (48) found that postoperative TMAO levels were higher than preoperative levels after bariatric surgery, which might be due to shortening of the small intestine and reduced anaerobic metabolism by the gut microbiota. Furthermore, some microbes that increase after RYGB, such as *Pseudomonas*, can convert trimethylamine (TMA) into TMAO *via* the enzyme TMA mono-oxidase, theoretically contributing to the higher TMAO levels (48). Other factors might also increase TMAO levels after RYGB surgery. As the generation of TMAO depends on oxidation by hepatic flavin-containing monooxygenase (FMO3), the improved hepatic steatosis seen after RYGB surgery might result in restored hepatic function and FMO3 activation. Furthermore, an *in vitro* study showed that FMO3 is inhibited by insulin (49). Thus, it is possible that, once insulin sensitivity has been restored, insulin levels decrease after RYGB surgery leading to higher expression of FMO3 and TMAO. Finally, dietary changes after RYGB surgery might affect TMAO levels, resulting in increased expression of carnitine and choline after initial diet-related reductions (50). Prospective studies with long-term follow-up are needed on the gut microbiota profile and microbiota-related metabolites in OSA. Altered diversity of the intestinal flora may be the cardinal factor in OSA and its associated cardiovascular, liver, and renal complications.

Indole-3-propionic acid is a bacterial metabolite derived from tryptophan in the gut that helps reduce the weight gain caused by antibiotics and a tryptophan-rich diet (51). The postoperative increase in indole-3-propionic acid promotes sustainable weight loss by attenuating the increased intestinal permeability and reversing the interferon gamma-induced transcriptional increase in expression of fructose transporter SLC2A5 (GLUT5) (52).

The level of C-peptide reflects the function of islet β-cells, and the decline of β-cell function is a prerequisite and core link for the onset of type 2 diabetes (53). With the increase in the severity of OSA, the islet function gradually declines, indicating that OSA is associated with impaired insulin sensitivity and function of islet β-cells (54). So, we hypothesis that a higher level of C-peptide before surgery suggesting a better islet function and a better compensate for the metabolic damage caused by OSA. Therefore, these subjects have better metabolic function and outcomes than those with lower C-peptide after TYGB, which is also of great significance to the improvement of OSA.

With the increasing prevalence of obesity and diabetes in the general population, OSA is also becoming increasingly common. Our results highlight the effect size of RYGB surgery for patients with obesity, T2DM and OSA, and demonstrate the metabolomic changes that occur after RYGB surgery. The main advantage of our procedure is the improvement of obesity and metabolic status associated with alleviation of OSA. This study is the first to explore the impact of RYGB surgery on the metabolomic profile of OSA patients. We established a simple model to predict the likelihood of OSA remission. However, several limitations of this study should be addressed. First, the study was conducted under limited clinical conditions with a relatively small sample size and an observational design. Further studies including larger populations and more distinct groups are necessary to validate our results, and structured investigations using cell and animal models are necessary to determine the underlying molecular mechanisms. Second, data on the duration of diabetes were not collected, which have affected the outcomes to some extent. Although metabolomics has potential for diagnosing and predicting the prognosis of OSA, studies are still in the preliminary stages. Results are not always consistent and clinical application remains some way off, such that currently used clinical assessment methods, including PSG, cannot yet be replaced. Metabolomic profiling can be affected by several variables such as gender, medications, nutrition, and diurnal variations, and additional studies are needed to clarify the biological functions of the metabolites identified herein.

## Conclusions

Our study demonstrated that RYGB surgery improved the metabolic status of OSA patients. Several preoperative indices in combination, i.e., age, the AHI and fasting C-peptide and hippurate levels, had high sensitivity and specificity for the prediction of OSA remission. A large population-based, prospective validation study of certain metabolites may help elucidate their potential roles as biomarkers of OSA remission.

## Authors Note

The English in this document has been checked by at least two professional editors, both native speakers of English. For a certificate, please see: http://www.textcheck.com/certificate/KmOQLp.

## Data Availability Statement

The raw data supporting the conclusions of this article will be made available by the authors, without undue reservation.

## Ethics Statement

The studies involving human participants were reviewed and approved by the Ethics Committee of Shanghai Jiao Tong University Affiliated Sixth People’s Hospital. The patients/participants provided their written informed consent to participate in this study.

## Author Contributions

The corresponding authors are responsible for the authenticity of the data. All authors made a significant contribution to the work reported, i.e., in the conception design or execution of the study, acquisition, analysis or interpretation of the data, or in all of these areas. WH, AZ, HX, XZ, HY, JG, and SY contributed to the study design, manuscript drafting or revision, or critical review of the article. HX, CX, AW, FW, XL, YL, JZ, and HZ took contributed to the data collection. WH, HX, XZ, CX, AW, FW, JZ, and HZ contributed to the statistical analyses. All authors approved the final version of the manuscript to be published, and agreed regarding the journal to which it has been submitted. All authors have agreed to be accountable for all aspects of the work.

## Funding

The study received grants from National Natural Science Foundation of China (Grant Nos. 82071030, 81700896, 81770988, 81970869), the Shanghai Municipal Commission of Science and Technology (Grant No. 18DZ2260200), Youth Science and Education Program of Suzhou, China (Grant No. KJXW2020018), Shanghai Science and Technology Innovation Program of Science and Technology Commission (Grant No. 20Y11902100), and Shanghai Shen-Kang Hospital Management Center Project (Grant Nos. SHDC2020CR2044B,SHDC2020CR3056B).

## Conflict of Interest

The authors declare that the research was conducted in the absence of any commercial or financial relationships that could be construed as a potential conflict of interest.

## Publisher’s Note

All claims expressed in this article are solely those of the authors and do not necessarily represent those of their affiliated organizations, or those of the publisher, the editors and the reviewers. Any product that may be evaluated in this article, or claim that may be made by its manufacturer, is not guaranteed or endorsed by the publisher.

## Supplementary Material

Supplementary Figure 1 Heatmap based on the Spearman correlation matrix demonstrated clear correlations between the clinical indicators and metabolomic profiles of all patients before and after RYGB. RYGB, Roux-en-Y gastric bypass surgery; SaO2, pulse oxygen saturation; BMI, body mass index; AHI, apnea–hypopnea index; ESS, Epworth sleepiness score; ODI, oxygen desaturation index; MAI, micro-arousal index; SBP, systolic blood pressure; DBP, diastolic blood pressure; FBG, fasting blood glucose; GHb, glycated hemoglobin; GA, glycated albumin; HOMA-IR, homeostasis model assessment of insulin resistance; TC, total cholesterol; TG, triglyceride; HDL, high-density lipoprotein cholesterol; LDL, low-density lipoprotein cholesterol; ApoA-I, apolipoprotein A-I; ApoB, apolipoprotein B; ApoE, apolipoprotein E; Lp(a), lipoprotein (a).

Supplementary Figure 2 Heatmap based on the Spearman correlation matrix demonstrated clear correlations between the clinical indicators and metabolomic profiles of OSA patients before and after RYGB. OSA, obstructive sleep apnea; RYGB, Roux-en-Y gastric bypass surgery; SaO2, pulse oxygen saturation; BMI, body mass index; AHI, apnea–hypopnea index; ESS, Epworth sleepiness score; ODI, oxygen desaturation index; MAI, micro-arousal index; SBP, systolic blood pressure; DBP, diastolic blood pressure; FBG, fasting blood glucose; GHb, glycated hemoglobin; GA, glycated albumin; HOMA-IR, homeostasis model assessment of insulin resistance; TC, total cholesterol; TG, triglyceride; HDL, high-density lipoprotein cholesterol; LDL, low-density lipoprotein cholesterol; ApoA-I, apolipoprotein A-I; ApoB, apolipoprotein B; ApoE, apolipoprotein E; Lp(a), lipoprotein (a).

Supplementary Figure 3 Heatmap based on the Spearman correlation matrix demonstrated clear correlations between the clinical indicators and metabolomic profiles of OSA remission patients before and after RYGB. OSA, obstructive sleep apnea; RYGB, Roux-en-Y gastric bypass surgery; SaO2, pulse oxygen saturation; BMI, body mass index; AHI, apnea–hypopnea index; ESS, Epworth sleepiness score; ODI, oxygen desaturation index; MAI, micro-arousal index; SBP, systolic blood pressure; DBP, diastolic blood pressure; FBG, fasting blood glucose; GHb, glycated hemoglobin; GA, glycated albumin; HOMA-IR, homeostasis model assessment of insulin resistance; TC, total cholesterol; TG, triglyceride; HDL, high-density lipoprotein cholesterol; LDL, low-density lipoprotein cholesterol; ApoA-I, apolipoprotein A-I; ApoB, apolipoprotein B; ApoE, apolipoprotein E; Lp(a), lipoprotein (a).

## Abbreviations

OSA, obstructive sleep apnea; T2DM, type 2 diabetes; RYGB, Roux-en-Y gastric bypass surgery; BMI, body mass index; CPAP, continuous positive airway pressure; PSG, polysomnography; SBP, systolic blood pressure; DBP, diastolic blood pressure; GHb, glycated hemoglobin; GA, glycated albumin; TC, cholesterol; TG, triglyceride; HDL, high-density lipoprotein; LDL, low-density lipoprotein; Apo, apolipoprotein; HOMA-IR, homeostasis model assessment of insulin resistance; Lp(a), lipoprotein (a); AHI, apnea-hypopnea index; LSpO2, lowest pulse oxygen saturation; SpO2, pulse oxygen saturation; ODI, oxygen desaturation index; MAI, microarousal index; ESS, Epworth Sleepiness Scale; AASM, American Academic Sleep Medicine; ROC, receiver operating characteristic; AUC, area under the curve; PCA, principal component analysis; PLS-DA, partial least squares-discriminant analysis; OPLS-DA, orthogonal partial least squares-discriminant analysis; VIP, variable importance in projection; CI, confidence interval; MUFA, monounsaturated fatty acid; TMAO, trimethylamine N-oxide; BCAA, branched chain amino acids; mTORC1, mammalian target of rapamycin complex; TMA, trimethylamine; FMO3, flavin-containing monooxygenase.
