# Supplementary material for: Metabolomics Analysis on Obesity-Related Obstructive Sleep Apnea After Weight Loss Management: A Preliminary Study
Source: Front Endocrinol (Lausanne). 2022 Jan 3;12:761547. doi: 10.3389/fendo.2021.761547 (PMC8761762; doi:10.3389/fendo.2021.761547)
Supplement: Supplementary Figure 1 — Heatmap based on the Spearman correlation matrix demonstrated clear correlations between the clinical indicators and metabolomic profiles of all patients before and after RYGB. RYGB, Roux-en-Y gastric bypass surgery; SaO2, pulse oxygen saturation; BMI, body mass index; AHI, apnea–hypopnea index; ESS, Epworth sleepiness score; ODI, oxygen desaturation index; MAI, micro-arousal index; SBP, systolic blood pressure; DBP, diastolic blood pressure; FBG, fasting blood glucose; GHb, glycated hemoglobin; GA, glycated albumin; HOMA-IR, homeostasis model assessment of insulin resistance; TC, total cholesterol; TG, triglyceride; HDL, high-density lipoprotein cholesterol; LDL, low-density lipoprotein cholesterol; ApoA-I, apolipoprotein A-I; ApoB, apolipoprotein B; ApoE, apolipoprotein E; Lp(a), lipoprotein (a). [file DataSheet_1.pdf]

Supplementary Material

1 Supplementary Figures

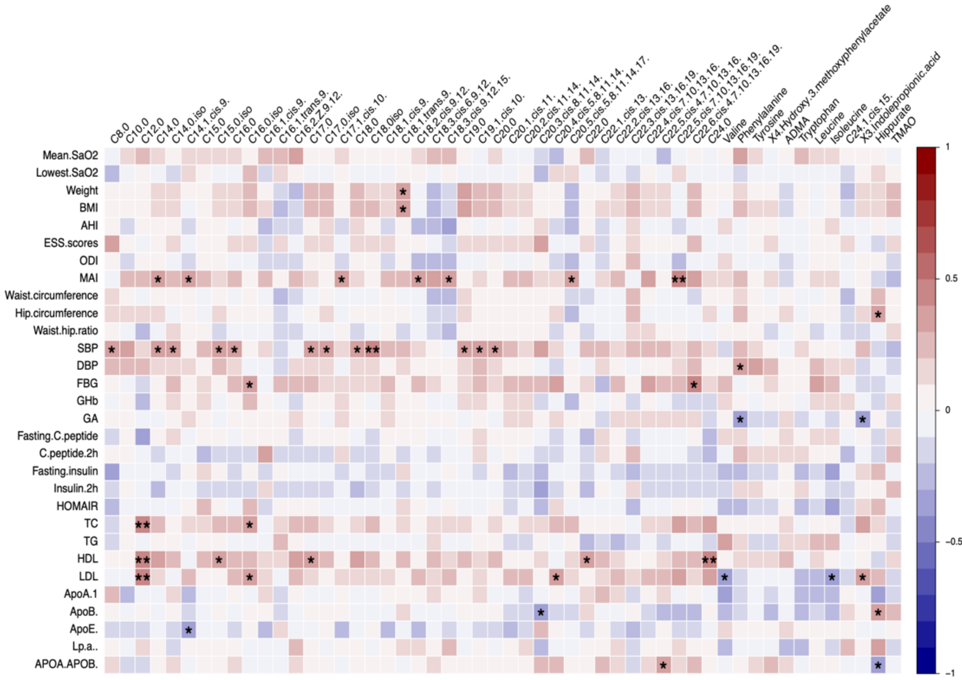

Supplementary Figure S1. Heatmap based on the Spearman correlation matrix demonstrated clear correlations between the clinical indicators and metabolomic profiles of all patients before and after RYGB.

Abbreviations: RYGB, Roux-en-Y gastric bypass surgery; SaO2, pulse oxygen saturation; BMI, body mass index; AHI, apnea–hypopnea index;

ESS, Epworth sleepiness score; ODI, oxygen desaturation index; MAI, micro-arousal index; SBP, systolic blood pressure; DBP, diastolic blood pressure; FBG, fasting blood glucose; GHb, glycated hemoglobin; GA, glycated albumin; HOMA-IR, homeostasis model assessment of insulin resistance; TC, total cholesterol; TG, triglyceride; HDL, high-density lipoprotein cholesterol; LDL, low-density lipoprotein cholesterol; ApoA-I, apolipoprotein A-I; ApoB, apolipoprotein B; ApoE, apolipoprotein E; Lp(a), lipoprotein (a).

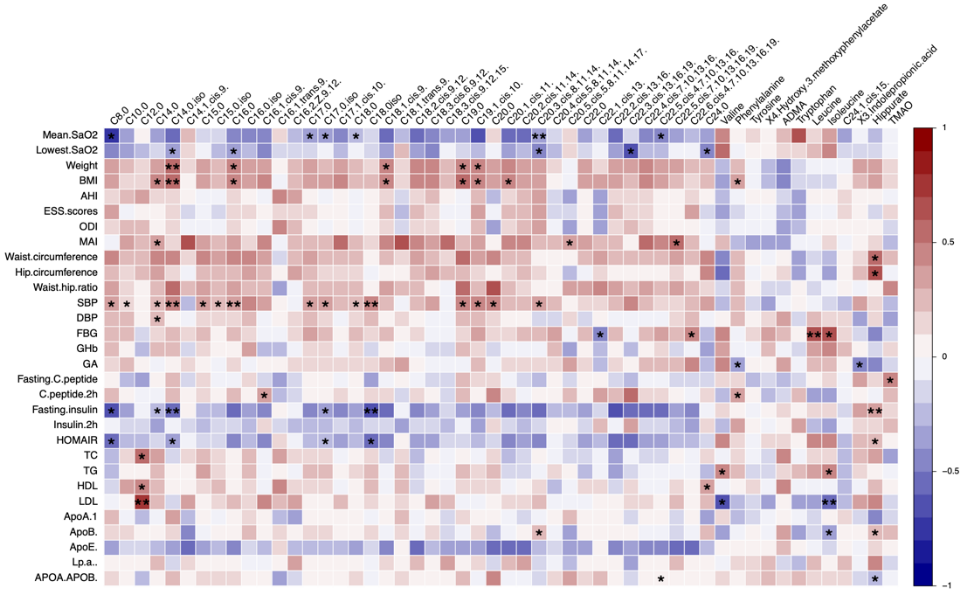

Supplementary Figure S2. Heatmap based on the Spearman correlation matrix demonstrated clear correlations between the clinical indicators and metabolomic profiles of OSA patients before and after RYGB.

Abbreviations: OSA, obstructive sleep apnea; RYGB, Roux-en-Y gastric bypass surgery; SaO2, pulse oxygen saturation; BMI, body mass index; AHI, apnea–hypopnea index; ESS, Epworth sleepiness score; ODI, oxygen desaturation index; MAI, micro-arousal index; SBP, systolic blood pressure; DBP, diastolic blood pressure; FBG, fasting blood glucose; GHb, glycated hemoglobin; GA, glycated albumin; HOMA-IR, homeostasis model assessment of insulin resistance; TC, total cholesterol; TG, triglyceride; HDL, high-density lipoprotein cholesterol; LDL,

low-density lipoprotein cholesterol; ApoA-I, apolipoprotein A-I; ApoB, apolipoprotein B; ApoE, apolipoprotein E; Lp(a), lipoprotein (a).

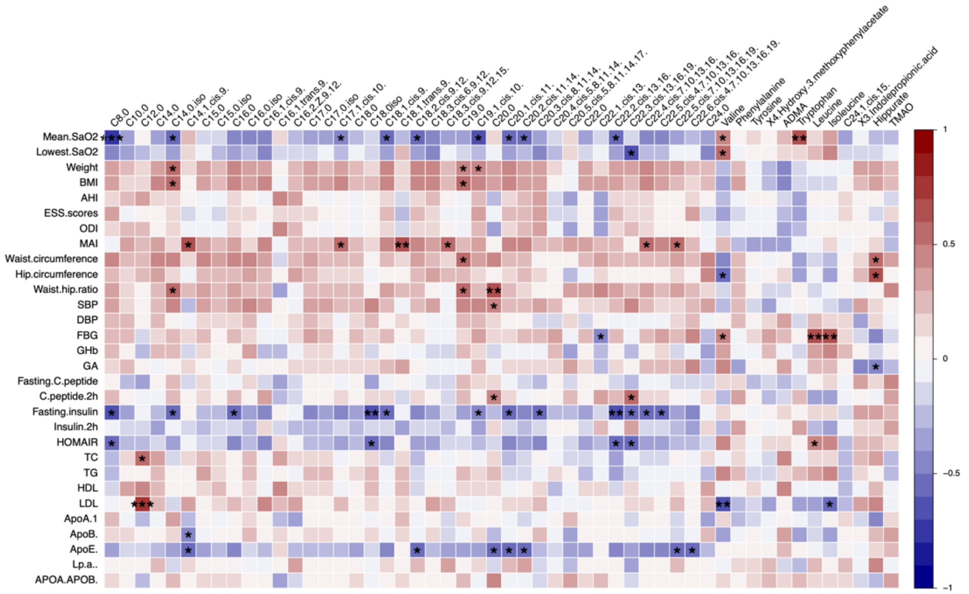

Supplementary Figure S3. Heatmap based on the Spearman correlation matrix demonstrated clear correlations between the clinical indicators and metabolomic profiles of OSA remission patients before and after RYGB.

Abbreviations: OSA, obstructive sleep apnea; RYGB, Roux-en-Y gastric bypass surgery; SaO2, pulse oxygen saturation; BMI, body mass index; AHI, apnea–hypopnea index; ESS, Epworth sleepiness score; ODI, oxygen desaturation index; MAI, micro-arousal index; SBP, systolic blood pressure; DBP, diastolic blood pressure; FBG, fasting blood glucose; GHb, glycated hemoglobin; GA, glycated albumin; HOMA-IR, homeostasis model assessment of insulin resistance; TC, total cholesterol; TG, triglyceride; HDL, high-density lipoprotein cholesterol; LDL, low-density lipoprotein cholesterol; ApoA-I, apolipoprotein A-I; ApoB, apolipoprotein B; ApoE, apolipoprotein E; Lp(a), lipoprotein (a).
