# Supplementary material for: Metabolomics Analysis on Obesity-Related Obstructive Sleep Apnea After Weight Loss Management: A Preliminary Study
Source: Front Endocrinol (Lausanne). 2022 Jan 3;12:761547. doi: 10.3389/fendo.2021.761547 (PMC8761762; doi:10.3389/fendo.2021.761547)
Supplement: Supplementary file 2 [file DataSheet_2.pdf]

Supplementary Material

2 Supplementary Tables

Supplementary Table S1. Clinical characteristics of the subgroup analyses with decrease rate of AHI after RYGB surgery

	Decrease rate of AHI after RYGB surgery ＞70％ (n = 17)		Decrease rate of AHI after RYGB surgery ≤ 70％ (n = 10)	
Characteristics	Before RYGB surgery	After RYGB surgery	Before RYGB surgery	After RYGB surgery
Males, n (％)	9 (52.94)		5 (50)	
Age, y	42.12±11.63		51.40±13.58	
Follow-up time, m	6.04±0.14		6.02±0.85	
Medication for T2DM, n (％)	17 (100)		9 (90)	
Medication for hypertension, n (％)	14 (82.35)		4 (40) [a]	
Medication for dyslipidaemia, n (％)	7 (41.18)		4 (40)	
Waist circumference, cm	108.65±14.67	86.45±9.24***	106.05±10.95	88.27±8.82***
Hip circumference, cm	110.31±10.48	96.98±7.36***	109.05±7.44	97.16±6.94**
Waist-to-hip ratio	0.98±0.07	0.89±0.05***	0.97±0.05	0.91±0.03**
BMI, kg/m^2	33.06±3.82	24.80±3.26***	31.90±3.77	25.02±3.12***
Decrease value of BMI, kg/m^2	8.26±1.97		6.88±1.96	
SBP, mmHg	137 (123-150)	120 (110-125)**	145 (129-162)	120 (118-130)***
DBP, mmHg	87 (80-97)	76 (70-82)	88 (80-99)	80 (70-85)***

Fasting glucose, mmol/L	7.76 (6.28-8.95)	5.11 (4.63-5.50)***	7.12 (6.21-8.87)	5.95 (5.36-6.41)** [b]
Fasting insulin, μU/mL	16.19 (11.73-30.92)	7.21 (4.20-9.69)***	21.55 (11.49-67.46)	8.53 (5.65-11.99)**
Insulin (120 min), μU/mL	75.80 (48.98-146.80)	13.50 (9.03-43.62)***	122.40 (52.12-158.53)	23.17 (14.01-36.00)**
Fasting C peptide, ng/mL	3.13 (2.72-4.37)	2.03 (1.59-2.46)***	3.03 (1.40-3.56) [a]	2.02 (1.71-2.38)
C peptide (120 min), ng/mL	9.10 (4.80-11.61)	4.91(3.53-7.11)**	4.96 (2.44-12.65)	4.23 (3.67-7.72)
HOMA-IR	5.60 (3.97-10.35)	1.65 (0.91-2.37)***	7.91 (3.32-21.11)	2.22 (1.20-3.51)**
GHb, %	7.30 (6.05-8.95)	5.50 (5.05-6.10)***	7.55 (6.33-9.45)	6.05 (5.40-6.70)**
GA, %	17.50 (14.15-19.75)	13.20 (11.45-14.40)***	17.36 (15.55-20.98)	15.15 (13.75-18.03)** [b]
TC, mmol/L	4.67 (3.97-5.10)	4.37 (3.87-4.85)	4.87 (4.32-5.66)	3.98 (3.51-4.40)***
TG, mmol/L	1.35 (1.22-2.60)	0.82 (0.72-1.19)**	1.67 (1.40-3.20)	1.05 (0.88-1.38)**
HDL, mmol/L	1.00 (0.90-1.06)	1.21 (1.11-1.32)***	1.07 (0.84-1.16)	1.13 (0.90-1.28)
LDL, mmol/L	2.98 (2.32-3.28)	2.58 (2.14-2.96)	2.88 (1.98-3.75)	2.16 (2.01-2.53)***
ApoA-1, g/L	1.06 (0.99-1.10)	1.07 (0.95-1.23)	1.11 (1.02-1.29)	1.08 (1.05-1.10)
ApoB, g/L	0.93 (0.90-0.94)	0.69 (0.57-0.73)***	0.90 (0.77-1.03)	0.69 (0.66-0.71)**
ApoE, mg/dL	5.12 (4.16-5.66)	3.66 (2.90-4.68)***	5.28 (4.72-6.52)	3.78 (3.54-4.73)**
Lp(a), mg/dL	16.47 (7.32-19.05)	16.00 (8.18-27.82)	14.70 (2.63-17.85)	16.60 (10.08-26.95)
ESS	8 (7-11)	3 (2-5)***	8 (3-10)	3 (2-6)**
ESS>10, n (%)	4 (23.53)	0***	2 (20.00)	0***
AHI, events/h	19.25 (12.00-29.55)	2.76 (1.60-2.88)***	23.97 (11.05-44.15)	8.85 (6.07-18.43)*** [b]
Decrease value of AHI, events/h	16.48 (10.20-25.70)		10.44 (2.98-22.13) [b]	

Decrease rate of AHI, %	85.64 (82.84-88.55)		64.84 (26.15-68.38) [b]	
LSpO2, %	80 (78-83)	88 (87-91)***	78 (67-83)	87 (83-90)**
Mean SpO2, %	94 (92-95)	96 (95-97)***	93 (92-95)	96 (94-97)**
ODI, events/h	21.55 (19.08-33.65)	5.00 (1.70-6.02)***	25.63 (12.68-44.30)	6.02 (1.50-17.63)
MAI, events/h	18.03 (13.15-17.92)	17.82 (9.05-18.89)	17.83 (14.48-20.63)	19.85 (16.42-27.40) [b]

Abbreviations: BMI, body mass index; SBP, systolic blood pressure; DBP, diastolic blood pressure; HOMA-IR, homeostasis model assessment of insulin resistance; GHb, glycated hemoglobin; GA, glycated albumin; TC, total cholesterol; TG, triglyceride; HDL, high-density lipoprotein cholesterol; LDL, low-density lipoprotein cholesterol; ApoA-I, apolipoprotein A-I; ApoB, apolipoprotein B; ApoE, apolipoprotein E; Lp(a), lipoprotein (a); ESS, Epworth sleepiness score; AHI, apnea–hypopnea index; LSpO2, lowest pulse oxygen saturation; SpO2, pulse oxygen saturation; ODI, oxygen desaturation index; MAI, microarousal index. * indicated p-value＜0.05 before and after surgery in subgroup analysis. ** indicated p-value＜0.05 before and after surgery in subgroup analysis. *** indicated p-value＜0.001 before and after surgery in subgroup analysis. [a] indicated the p-value for the difference between the 2 groups before surgery. [b] indicated the p-value for the difference between the 2 groups after surgery.

Supplementary Table S2. Difference in baseline metabolites levels between the OSA remission and non-remission groups

Class	Metabolite	B.Mean	C.Mean	B.Median	C.Median	B.SD	C.SD	B.IQR	C.IQR	P	FDR	FC	log2FC	test.method
SFAs	C8:0	0.219842283	0.283159704	0.178484851	0.207193611	0.147948318	0.194108943	[0.14064572375,0.3366509775]	[0.161209161,0.458221469]	0.459621468	0.912363757	1.288012934	0.36514708	t.test
SFAs	C10:0	0.06668018	0.061911118	0.070035608	0.050866751	0.027904857	0.025612122	[0.03899684375,0.08794648725]	[0.04324687775,0.07358253075]	0.71110496	0.912363757	0.928478568	-0.107059485	t.test
SFAs	C12:0	0.287615566	0.320313804	0.30397205	0.324047358	0.07135556	0.095719121	[0.23343410025,0.33012676075]	[0.2689670615,0.37766059525]	0.436290592	0.907484431	1.113687306	0.155344219	t.test
SFAs	C14:0	8.367677846	9.081422499	8.016931435	8.688080138	1.620070944	1.984522818	[7.547825525,8.968226042]	[7.60226536725,10.4773039325]	0.425624583	0.907484431	1.085297817	0.118090987	t.test
Unknown	C14:0 iso	0.133394589	0.169568756	0.151783983	0.176616303	0.041621111	0.043364838	[0.1222022015,0.15747786275]	[0.13436875725,0.21194192]	0.145710499	0.907484431	1.163603037	0.218598967	wilcox.test
MUFAs	C14:1(cis-9)	0.243944809	0.683376773	0.230976894	0.294789629	0.103896236	1.002588201	[0.17447155,0.312851554]	[0.17736877625,0.57027280225]	0.359934183	0.907484431	1.276273243	0.351937236	wilcox.test
SFAs	C15:0	1.129489887	1.30261002	1.152414508	1.139608446	0.142008327	0.54925836	[1.019942211,1.17794423675]	[1.0302774755,1.42621706725]	0.828557064	0.912363757	0.988887626	-0.016121508	wilcox.test
Unknown	C15:0 iso	0.8080218	0.857809113	0.760712471	0.791435366	0.117108981	0.195918644	[0.72432939425,0.90167545375]	[0.75019767275,1.01910220175]	0.539265511	0.912363757	1.061616299	0.086262426	t.test
SFAs	C16:0	53.96803789	61.49006099	56.92250485	62.04443113	11.85525394	23.24462704	[46.9627721325,60.2679293125]	[45.2254535275,72.66629334]	0.424659833	0.907484431	1.139379221	0.188248001	t.test
Unknown	C16:0 iso	0.471913734	0.565360025	0.470597951	0.548084487	0.049736585	0.174641896	[0.45428019325,0.489006295]	[0.4373860495,0.6557860685]	0.359934183	0.907484431	1.164655488	0.219903261	wilcox.test
MUFAs	C16:1(cis-9)	3.057411132	2.697596739	2.361339917	2.2029929	1.635573957	1.655566251	[1.9803975265,4.0990697845]	[1.7549949665,3.48324742175]	0.633438457	0.912363757	0.932941879	-0.100140888	wilcox.test

MUFAs	C16:1(trans-9)	0.301702858	0.195198151	0.200922922	0.180634229	0.244725869	0.068946548	[0.172549709,0.4176992945]	[0.153459546,0.20952335625]	0.31541661	0.907484431	0.899022504	-0.153570865	wilcox.test
Unknown	C16:2(Z-9,12)	0.064210193	0.084947641	0.067056768	0.076994674	0.018337776	0.058609006	[0.0550107925,0.07338509975]	[0.0600184655,0.08526395275]	0.408245349	0.907484431	1.148201388	0.199375704	wilcox.test
SFAs	C17:0	2.934204071	3.196360499	2.919030197	3.076047538	0.264444166	0.642936145	[2.871856208,2.9899272045]	[2.768581267,3.5975064005]	0.408245349	0.907484431	1.053790927	0.075588664	wilcox.test
Unknown	C17:0 iso	2.121049239	2.302077843	2.080071571	2.161675634	0.249833652	0.502987779	[2.00394629325,2.19706586]	[1.9388623625,2.70144505975]	0.37469888	0.907484431	1.08534861	0.118158506	t.test
MUFAs	C17:1(cis-10)	0.267133864	0.254562038	0.229911149	0.248546241	0.125797837	0.109793435	[0.19664852725,0.2534094395]	[0.1761912965,0.33724172525]	0.965400612	0.965400612	1.081053451	0.112437857	wilcox.test
SFAs	C18:0	61.72705615	74.07690482	61.13019916	72.38401414	12.96424168	27.49024403	[54.0674700325,63.00613791]	[58.34181924,92.3497904975]	0.236985237	0.907484431	1.184095834	0.243785849	wilcox.test
Unknown	C18:0iso	0.255073303	0.273521341	0.255779285	0.278844645	0.030715726	0.073733667	[0.241113843,0.266232036]	[0.21560042275,0.33463660425]	0.633438457	0.912363757	1.09017681	0.124562137	wilcox.test
MUFAs	C18:1(cis-9)	23.80537525	27.26532482	19.67251068	24.32800413	9.779794954	14.08004787	[18.9485802675,26.0508841475]	[16.639673905,32.7061414125]	0.828557064	0.912363757	1.236649685	0.306436875	wilcox.test
Unknown	C18:1(trans-9)	0.178010716	0.167716385	0.173253212	0.172294773	0.043055298	0.046575558	[0.14409733225,0.21814754325]	[0.134997203,0.20866806725]	0.637118114	0.912363757	0.94217016	-0.085940455	t.test
PUFAs	C18:2(cis-9,12)	25.29447286	32.23677463	24.02713946	31.55915376	8.485422053	15.46038868	[19.11410578,29.6842500425]	[18.43081616,45.62484557]	0.514785868	0.912363757	1.313479443	0.393393622	wilcox.test
PUFAs	C18:3(cis-6,9,12)	0.545515839	0.61728291	0.611355004	0.569055706	0.204365453	0.316669115	[0.36645964525,0.6778930015]	[0.3498879625,0.8357743995]	0.589463868	0.912363757	1.13155818	0.178310764	t.test
PUFAs	C18:3(cis-9,12,15)	1.853959452	1.935573345	2.024461745	1.652981445	0.651919873	0.871222611	[1.29139205675,2.29597774375]	[1.345655817,2.48687783625]	0.829221874	0.912363757	1.044021401	0.062151286	t.test
SFAs	C19:0	0.3407	0.3820	0.3273	0.3663	0.0464	0.1047	[0.30699174325,0.	[0.309858658,0.44	0.4082	0.9074	1.1191	0.16240	wilcox.test

		57874	28809	12051	1286	38232	28683	35760567425]	871854375]	45349	84431	54823	9632	
MUFAs	C19:1(cis-10)	0.032121688	0.02899075	0.031427283	0.027003008	0.010823078	0.020370289	[0.02389842,0.0389077735]	[0.01599660325,0.05040730125]	0.702723364	0.912363757	0.902528854	-0.147955038	t.test
SFAs	C20:0	0.938281171	0.983011191	0.911181246	0.962923096	0.103976075	0.203373615	[0.8501309975,1.034589034]	[0.843566807,1.1173031055]	0.5841679	0.912363757	1.047672299	0.067187527	t.test
MUFAs	C20:1(cis-11)	0.409379557	0.453335611	0.350015229	0.465268659	0.121130132	0.169444722	[0.3199973875,0.512824131]	[0.37740008425,0.5067576505]	0.547744629	0.912363757	1.107372372	0.147140433	t.test
PUFAs	C20:2(cis-11,14)	0.676686159	0.826097636	0.680719861	0.833517044	0.181382944	0.262519131	[0.5631125965,0.7493751155]	[0.65940277475,1.021834008]	0.195947505	0.907484431	1.220798778	0.287825423	t.test
PUFAs	C20:3(cis-8,11,14)	5.968900958	5.960522001	5.574011715	6.301634146	3.656722339	2.73797691	[3.4554211855,6.61404034775]	[4.03717068275,6.74236911125]	0.896750309	0.951653389	1.130538375	0.177009964	wilcox.test
PUFAs	C20:4(cis-5,8,11,14)	4.009896135	4.150965235	3.775901506	3.93060906	0.871645326	0.749222954	[3.43137940325,4.27851629825]	[3.607470279,4.52914463625]	0.716990749	0.912363757	1.035180238	0.049881981	t.test
PUFAs	C20:5(cis-5,8,11,14,17)	0.736903268	0.616852252	0.682993633	0.640220799	0.355594306	0.22077402	[0.44106110475,0.873642225]	[0.41933397125,0.74293898725]	0.394107009	0.907484431	0.837087144	-0.256550274	t.test
SFAs	C22:0	0.027031396	0.026886494	0.026531569	0.027523239	0.005092379	0.007543123	[0.02469669125,0.031018387]	[0.02229942225,0.030644655]	0.963679112	0.965400612	0.994639468	-0.007754416	t.test
MUFAs	C22:1(cis-13)	0.061208245	0.092598886	0.049530033	0.041704386	0.040527053	0.127242636	[0.03044972275,0.080987807]	[0.03108617,0.07294232925]	0.761826409	0.912363757	0.842001991	-0.248104449	wilcox.test
Fatty Acids	cis-13,16-Docosadienoic acid	0.018111546	0.025648143	0.018891259	0.026259083	0.004895666	0.009169255	[0.01716870725,0.020278676]	[0.019439215,0.03270903825]	0.061906441	0.906204906	1.416121145	0.501944689	t.test
PUFAs	C22:3(cis-13,16,19)	0.014356778	0.013548262	0.014708826	0.011483285	0.006884836	0.009413901	[0.009803551,0.0190048085]	[0.006333661,0.01790847925]	0.842181929	0.912363757	0.943683969	-0.083624299	t.test
PUFAs	C22:4(cis-7,10,13,16)	0.242267144	0.266111228	0.229781818	0.260188888	0.069140026	0.085986661	[0.21617354125,0.2654179125]	[0.19772306475,0.3219101255]	0.535064044	0.912363757	1.098420626	0.135430621	t.test

PUFAs	C22:5(cis-4,7,10,13,16)	0.18786546	0.227329456	0.182371319	0.198148909	0.067940588	0.077474992	[0.1539777805,0.2054733975]	[0.1658574045,0.28157026025]	0.275847357	0.907484431	1.210065203	0.275084788	t.test
PUFAs	C22:5(cis-7,10,13,16,19)	0.999819611	0.975461008	0.858984962	0.96154968	0.460324115	0.291743532	[0.6608177135,1.201285147]	[0.75926535225,1.1363541195]	0.761826409	0.912363757	1.119402228	0.162728524	wilcox.test
PUFAs	C22:6(cis-4,7,10,13,16,19)	11.9748442	11.37751068	11.62388919	11.81857363	4.491619123	3.926247785	[8.6239885595,15.404092475]	[8.884897678,13.94348212]	0.767507599	0.912363757	0.950117638	-0.073821945	t.test
SFAs	C24:0	0.016670135	0.017013325	0.016773871	0.015613942	0.008129396	0.003598168	[0.0102762805,0.02292730225]	[0.01527427325,0.01889237025]	0.828557064	0.912363757	0.930849085	-0.103380807	wilcox.test
MUFAs	C24:1(cis-15)	0.01176302	0.015365538	0.010578099	0.016784074	0.002436697	0.004372975	[0.01011064325,0.01326903475]	[0.01237719175,0.0176860125]	0.062532705	0.906204906	1.306257961	0.38543983	t.test
Organic nitrogen compounds	TMAO	0.338133475	0.681841486	0.302452278	0.573056931	0.198842521	0.415454084	[0.20323938175,0.47332593325]	[0.32711353925,1.14017593]	0.121989122	0.906204906	1.894701982	0.921970944	wilcox.test
Amino Acids	Valine	58.00776541	53.93054174	58.17449573	57.07467792	8.677671796	10.37390444	[50.8075765525,62.088329355]	[50.5139381375,58.2188429]	0.388785738	0.907484431	0.929712451	-0.105143519	t.test
Amino Acids	Leucine	0.604338186	0.466022222	0.621635931	0.466014256	0.176623116	0.135695273	[0.455797548,0.73693640275]	[0.41233405775,0.50669753825]	0.078663574	0.906204906	0.771128207	-0.374957353	t.test
Amino Acids	Isoleucine	4.998771118	4.319847212	4.908054619	4.544847535	1.104660975	1.017428493	[3.99879239425,5.79405234225]	[4.27489532425,4.7903818635]	0.195000441	0.907484431	0.864181838	-0.210593184	t.test
Amino Acids	Phenylalanine	2.256913688	2.077463677	2.136570023	2.21008057	0.444210752	0.675749054	[2.011544708,2.5853330235]	[1.72468181575,2.567644307]	0.52992629	0.912363757	0.920488758	-0.119527993	t.test
Amino Acids	Tyrosine	0.58052385	0.655187267	0.587698535	0.69922982	0.101461631	0.173719222	[0.4969618185,0.64526728]	[0.5296697965,0.80044477925]	0.304914074	0.907484431	1.128613866	0.17455198	t.test
Unknown	4-Hydroxy-3-methoxyphenylacet	0.446309373	0.641458243	0.442007854	0.6208182	0.125970749	0.295062903	[0.36344604825,0.5666096825]	[0.530078203,0.88533827925]	0.101101513	0.906204906	1.404541107	0.490098849	wilcox.test

	ate													
Amino Acids	Asymmetric Dimethylarginine	0.1983216	0.186269119	0.201501715	0.185825954	0.060956878	0.053919946	[0.147725176,0.24216088675]	[0.15691346875,0.2080253945]	0.662667089	0.912363757	0.939227594	-0.0904533	t.test
Amino Acids	Tryptophan	11.70068227	9.681186123	10.73711046	9.862774311	2.922314697	2.285454202	[10.4522663525,13.2132624275]	[8.153906542,10.80371948]	0.119444502	0.906204906	0.827403556	-0.273336936	t.test
Benzene and substituted derivatives	Hippurate	0.266051959	0.90685541	0.146002182	0.524027696	0.32445142	0.834889816	[0.0905281395,0.30581456925]	[0.39763193425,1.492823097]	0.043420632	0.906204906	3.58917715	1.843653132	wilcox.test
Indoles	Indole-3-propionic acid	0.115508642	0.149447624	0.110187937	0.123006735	0.054030623	0.151453703	[0.08280083025,0.15893278125]	[0.03570407475,0.191625735]	0.965400612	0.965400612	1.116335766	0.158771019	wilcox.test

Abbreviations: OSA, obstructive sleep apnea; SD, standard deviation; IQR, interquartile range; FDR, false discovery rate; FC, fold change; SFA, saturated fatty acids; MUFA, monounsaturated fatty acid; PUFA, polyunsaturated fatty acids; TMAO, trimethylamine N-oxide.

B indicated OSA remission group and C indicated OSA non-remission group.

Supplementary Table S3. Metabolite levels of patients with OSA remission before and after RYGB surgery

Class	Metabolite	B.Mean	E.Mean	B.Median	E.Median	B.SD	E.SD	B.IQR	E.IQR	P	FDR	FC	log2FC	test.method
Amino Acids	Valine	58.00776541	46.84316	58.17449573	47.82098702	8.677671796	6.567443929	[50.8075765525,62.088329355]	[45.3422573025,49.6614244725]	0.002879473	0.07486631	0.82202667	-0.282742893	wilcox.test
Amino Acids	Isoleucine	4.998771118	4.015072696	4.908054619	3.937358816	1.104660975	0.919534749	[3.99879239425,5.79405234225]	[3.56121956625,4.612895483]	0.044598307	0.38651866	0.80321195	-0.316147362	t.test
MUFAs	C24:1(cis-15)	0.01176302	0.007642282	0.010578099	0.008081388	0.002436697	0.004858207	[0.01011064325,0.01326903475]	[0.00341517375,0.0095979185]	0.031897015	0.331728959	0.64968705	-0.622183147	t.test
Organic nitrogen compounds	TMAO	0.338133475	1.18939251	0.302452278	0.971778655	0.198842521	0.919618496	[0.20323938175,0.47332593325]	[0.4746948265,1.814554108]	0.023230639	0.301998311	3.2129983	1.683920217	wilcox.test
Benzene and substituted derivatives	Hippurate	0.266051959	0.920874762	0.146002182	0.856297508	0.32445142	0.556331573	[0.0905281395,0.30581456925]	[0.50285306475,1.4200048375]	0.002089242	0.07486631	5.864963768	2.552122196	wilcox.test
Indoles	Indole-3-propionic acid	0.115508642	0.238367065	0.110187937	0.215916792	0.054030623	0.13317995	[0.08280083025,0.15893278125]	[0.14834825025,0.2697217265]	0.014689645	0.254620508	1.95953204	0.970509162	wilcox.test

Abbreviations: OSA, obstructive sleep apnea; RYGB, Roux-en-Y gastric bypass surgery; SD, standard deviation; IQR, interquartile range; FDR, false discovery rate; FC, fold change; MUFA, monounsaturated fatty acid; TMAO, trimethylamine N-oxide.

B indicates preoperative OSA remission group and E indicates postoperative OSA remission group

Supplementary Table S4. Adjusted ORs and 95% CIs for the associations of parameters with OSA remission

Predictors	n	OR (95% CI)		
		Model 1	Model 2	Model 3
age	27	0.874 (0.784–0.975)*	0.844(0.739–0.963)*	0.727 (0.377–1.401)
AHI	27	0.920 (0.855–0.991)*	0.912 (0.844–0.985)*	1.092 (0.732–1.630)
fasting C-peptide level	27	3.201 (1.085–9.443)*	3.726 (1.162–11.953)*	12.919 (0.445–375.323)
hippurate level	27	0.131 (0.013-1.281)	0.123 (0.011–1.422)	0.103 (0.006–1.802)

Model 1 was adjusted for BMI, and sex; Model 2 was adjusted for variables included in Model 1 and fasting glucose, fasting insulin; Model 3 was adjusted for variables included in Model 2 and medication for T2DM, hypertension and dyslipidaemia，ESS, LSpO2. Logistic regression was used to further analyze the data. Abbreviations: OR, odds ratio; CI, confidence interval; OSA, obstructive sleep apnea; AHI, apnea-hypopnea index; BMI, body mass index; T2DM, type 2 diabetes; ESS, Epworth Sleepiness Scale; LSpO2, lowest pulse oxygen saturation. *p indicated a significant difference.
